# Arginine‐rich membrane‐permeable peptides are seriously toxic

**DOI:** 10.1002/prp2.334

**Published:** 2017-08-24

**Authors:** Qian Li, Mengchuan Xu, Yali Cui, Chunqian Huang, Manji Sun

**Affiliations:** ^1^ State Key Laboratory of Toxicology and Medical Countermeasures Institute of Pharmacology and Toxicology Military Academy of Medical Sciences Beijing China

**Keywords:** Arginine, arginine‐rich peptides, membrane‐permeable peptides

## Abstract

The membrane‐permeable peptides (MPP) such as undecapeptides TAT (YGRKKRRQRRR) and CTP (YGRRARRRRRR) have been receiving much attention for delivering various kinds of low membrane‐permeability materials in vitro and in vivo. We have successfully used MPP in carrying various proteins through blood‐brain barrier (BBB) in treatment of many kinds of nervous diseases. However, people always concentrate their mind on the efficacy and the mechanism of permeation of the conjugates across BBB, but overlook the toxicity of the membrane‐permeable peptide itself. Once we injected intravenously not very large amounts of gamma‐aminobutyric acid‐MPP (GABA‐MPP) to the mice, to our great surprise, the mice died within seconds with seizure, whereas the GABA control mice well survived. Thus, the importance of the toxicity of MPPs and their conjugates comes into the field of our vision. The low LD_50_ values of arginine‐rich TAT (27.244 mg kg^−1^) and CTP (21.345 mg kg^−1^) *per se* in mice indicate that they all fall within the range of highly toxic chemicals. Among the arginine‐rich peptides, R11 (RRRRRRRRRRR), a peptide composed purely of arginine residues, has the lowest LD_50_ value (16.5 mg kg^−1^) and manifests the highest toxicity, whereas TD (ACSSSPSKHCG), a peptide without arginine residue, shows a much lower toxicity and higher survival rate in mice. The mass percentage of arginine‐rich MPP in the conjugate is critically important, the mass radio of arginine in the MPP appears a linear correlation with the toxicity. Thus we conclude, the arginine‐rich MPPs are more suitable for using in the macro‐molecular conjugates, but not in the small‐molecular one.

**Abbreviations**
GABA: *γ*‐aminobutyric acid
MPP: membrane‐permeable peptides
SPSS: statistical package for the social science
TFA: Trifluoroacetic acid

## Introduction

The arginine‐rich membrane‐permeable peptides (MPP) such as the undecapeptides TAT (YGRKKRRQRRR) and CTP (YGRRARRRRRR) have been widely used in many laboratories for carrying various kinds of exogenous bio‐molecules including peptides, proteins, nucleotides and nucleic acids to penetrate across different kinds of bio‐membranes in vitro and in vivo. However, people always concentrate their mind on the efficacy and the mechanism of permeation of the conjugates, but overlook the toxicity of the membrane‐permeable peptide itself. Once we injected intravenously not very large amounts of *γ*‐aminobutyric acid ‐TAT (GABA‐TAT) to the mice, to our great surprise, the mice died within seconds with seizure. Thus in this paper, the toxicity of MPPs *per se* was attentively studied.

## Materials and Methods

### Animals

A total of 390 mice (Kunming species, 22 ± 2 g, sex matched), were provided by the Experimental animal center of Academy of military medical sciences, China. The health condition of the mice was examined to confirm their suitability for use in the study. The animal rooms were monitored and maintained under a 12 h light‐dark cycle with temperature ranging from 20 to 25°C and a relative humidity of 40–60%. All the mice were allowed to acclimate to the laboratory environment for 2 days, housed by sex in groups of five per cage, provided with standard commercial diet and drinking water ad libitum. All the animal procedures performed in this study were reviewed and approved by the Animal Experimental Welfare and Ethical Inspection Committee of the Chinese Center for Disease Control and Prevention (Approval number IACUC#15‐026).

### Reagents

The synthetic peptides TAT (YGRKKRRQRRR), CTP (YGRRARRRRRR), R11 (RRRRRRRRRRR), TD (ACSSSPSKHCG), and their GABA‐conjugates were synthetized by SBS Genetech Co, Ltd. The peptide molecular weights were confirmed by mass spectrometry, 98% in purity. The lyophilized peptides were prepared and diluted in 0.9% NaCl before use.

*γ*‐aminobutyric acid (GABA) was provided by Beijing Gleckes Bio‐engineering Technology Co. Ltd. with a purity of 99%.

TAT‐choline acetyltransferase (TAT‐ChAT): The recombinant prokaryotic plasmid of pET15‐TAT‐ChAT was constructed to express the TAT‐ChAT fusion protein in *Escherichia coli BL21* based on previous works (Fu et al. 2004, 2005). The purified TAT‐ChAT fusion protein (98% in purity) could effectively ameliorate the dementia symptom of the aged mice and the APP/PS1‐TgN transgenic mice in our other experiments. Lyophilized TAT‐ChAT was stored at −20°C, and dissolved in 0.9% saline solution before use.

Trifluoroacetic acid (TFA) was purchased from ACROS Organics (Belgian), extra pure with a purity of 99%. Formulated solutions (0.1%,0.5%,1%,2%,5%) were diluted with sterilized deionized water.

### Experimental design

Firstly, a preliminary sequential toxicity testing was carried to find out the scope of the dosage. Then four dosages within this range were properly selected to carry on the LD_50_ estimation. The LD_50_ was calculated avoid the all or none survival groups of mice.

#### Experimental procedures

Five males and five females per group were stratified by weight and assigned randomly. The control mice and those in groups 1, 2, 3, 4 were intravenously injected with different doses of MPPs or MPP‐conjugates. The general behavior and signs of toxicity were monitored for 3 h after administration, and carried on once a day up to 14 days. The surviving mice were euthanized while those died from the poisoning were necropsied.

### Statistical analysis

All the data are expressed as the means ± standard deviation (SD), and comparisons among the different groups were performed using an analysis of variance (ANOVA). The IBM statistical package for the social science (SPSS) statistics 20 software was used for all the analysis. The significance level was set at 5 and 1% (*P *<* *0.05 and *P *<* *0.01). The LD_50_ value was determined according to the Bliss method (Badisa et al. 2008).

## Results

### LD_50_ of MPPs and their GABA conjugates

Firstly, we found that TAT or CTP injected *via* the tail vein were highly toxic to the mice (Table 1). The low LD_50_ values of TAT (27.244 mg kg^−1^) and CTP (21.345 mg kg^−1^) indicate that they all fall within the range of highly toxic chemicals (Table 2). GABA is not toxic at all even at dose as high as 8000 mg kg^−1^. However, when TAT and CTP were, respectively, conjugated with GABA and intravenously injected to mice, the toxicity of the conjugates became even higher than that of TAT or CTP alone (Table 3). The mice immediately jumped up, kicked the hind‐limbs, and manifested bug‐eyes and abdominal respiration. All the mice were difficult to turn‐over the body and died within one minute.

**Table 1:** Weight ratios of the membrane‐permeable peptides in their conjugates (mice,iv injection. aa denotes the amino acid residue)

	Dosage(mg kg^−1^)	Survival rate (%)	Molecular weight (Dalton)	Weight of MPP in conjugates (%)	References
GABA	8000	100	103	0	
TD (11aa)	140	100	1065	100	
TAT(11aa)	27	50	1560	100	
CTP(11aa)	21	50	1558	100	
R11(11aa)	16.5	50	1735	100	
GABA –TD	100	100	1149	92.7	
GABA –TAT	19	50	1645	94.8	
GABA –CTP	13	50	1644	94.8	
TAT –ChAT	339	50	70000	2.2	
TAT –hEGF	0.1	100	7000	22.3	Zhao et al. (2011a)
TAT –BDNF	5	100	14400	10.8	Zhou et al. (2008);
TAT –tCNTF	1	100	21664	7.2	Qu et al. (2008)
TAT ‐P53	5	100	54000	2.9	Zhao et al. (2009); Zhao et al. (2011b)
TAT ‐TH	8	100	61000	2.6	Wu et al. (2006); Fu et al. (2006);
TAT –ChAT	4	100	70000	2.2	Fu et al. (2004, 2005)

MPP, membrane‐permeable peptides; GABA, *γ*‐aminobutyric acid.

**Table 2:** Mortality of mice in acute toxicity study (*n* = 10)

MPPs	LD50 (mg kg^−1^)	Groups	Control group	1	2	3	4
CTP	21.345	Dose (mg kg^−1^)	0	19.5	20	20.5	22
		Mortality	0/10	2/10	3/10	4/11	6/10
		Mortality (%)	0	20	30	36.4	60
TAT	27.244	Dose (mg kg^−1^)	0	25	27	28	30
		Mortality	0/10	2/9	4/9	5/9	7/8
		Mortality (%)	0	22.2	44.4	55.6	87.5
R11	16.508	Dose (mg kg^−1^)	0	15.5	16	16.5	17
		Mortality	0/10	1/10	3/10	5/10	7/10
		Mortality (%)	0	10	30	50	70
TD	>140	Dose (mg kg^−1^)	0	80	100	140	–
		Mortality	0/10	0/2	0/8	0/8	–
		Mortality (%)	0	0	0	0	–

MPPs, membrane‐permeable peptides.

**Table 3:** Mortality and survival periods of mice after injection of GABA‐MPPs

GABA‐MPPs	Dose (mg kg^−1^)	Mortality	Survival time (min)
GABA‐CTP	≤10	0/2	Normal
	12.69	0/2	>180
	13.05	2/5	2, 4
	13.41	4/4	1, 2, 1, 1
	≥15	2/2	<1
GABA‐TAT	≤13.41	0/2	Normal
	19.1	2/4	>180
	19.2	3/4	2, 3, 4
	19.3	3/4	1, 2, 1
	≥19.5	2/2	1, 1
GABA‐TD	50	0/4	Normal
	100	0/4	Normal

MPPs, membrane‐permeable peptides; GABA, *γ*‐aminobutyric acid.

We also considered whether there was some residual TFA (<0.1%) in the synthetic peptides, thus we set a series of TFA control groups in the experiments. It showed that 0.1, 0.5 and 1% of TFA did not manifest any toxicity, whereas the dose rose up to 2%, death appeared in a part of the mice. It seems that residual TFA if any in the synthetic peptides does not influence the results obtained in our experiments.

### LD_50_ of TAT‐ChAT

The LD_50_ and LD_99_ of TAT‐CHAT in mice came to 338.84 and 480 mg kg^−1^, respectively, (95% of confidence interval was between 275.42 and 416.87 mg kg^−1^). It agrees with the results obtained in many macro‐molecular protein conjugates that we have estimated (Fu et al. 2004, 2005, 2006; Wu et al. 2006; Qu et al. 2008; Zhou et al. 2008; Zhao et al. 2009, 2011a,b). In view of the great difference between the small molecule conjugates and the large molecule conjugates, it seems that the mass ratio of MPP in the conjugate is the key to the question. The larger the mass ratio of MPP in the conjugate, the more toxic the conjugate will be (Table 1).

### Toxicity of arginine

The mechanism of the poisonous effects caused by TAT and CTP is not yet well understood. However, it is known that L‐arginine stimulates the release of insulin and causes necrotic pancreatitis with specific elevation of amylase in serum and urine. The LD_50_ of arginine in rats come to 3793 mg kg^−1^ (corresponding to 542 mg kg^−1^ in mice) (Saka et al. 2004). Thus, the higher mass ratio of arginine residues in the conjugate (Table 4) might be the best explanation of the toxicity.

**Table 4:** Amino acid sequences of the membrane‐permeable undecapeptides

Peptides	Sequence	Molecular weight (Dalton)	Content of Arginine (%)	LD_50_ (mg kg^−1^)
TAT	YGRKKRRQRRR	1560	61	27
CTP	YGRRARRRRRR	1558	81	21
R11	RRRRRRRRRRR	1735	100	16.5
TD	ACSSSPSKHCG	1065	0	>140

For a better understanding of the action of arginine, we tested a undecapeptide (R11) completely composed of arginine residues, and another undecapeptide (TD) without any arginine residue. When TD was injected to mice at dose of 140 mg kg^−1^ *via* the tail vein, all the mice well survived even without any side effect appeared, and so did for its GABA conjugate at dose of 100 mg kg^−1^ (Table 1). The toxicity of TD is much less than that of TAT and CTP and has a higher survival rate in the in vivo experiments (Table 1 and 2). On the other hand, R11 has a much lower LD_50_ value than that of TAT, CTP and TD. It clearly indicates that transmembrane delivery in vivo *via* the phage displayed peptide TD is an ideal non‐invasive membrane‐permeable carrier.

**Figure 1:**
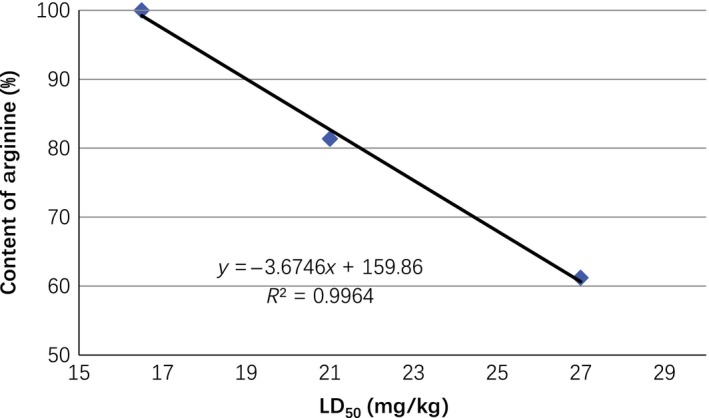
The arginine residue contents in the membrane‐permeating conjugates are correlated with the toxicity to the mice.

All these results further support the above‐mentioned explanation of the toxicity of arginine in MPPs. The arginine residue contents in the membrane‐permeating conjugates are correlated with the toxicity to the mice, and the contents of arginine in MPPs calculated based on the relative molecular mass of arginine (157 Dalton) are linear correlation with the toxicity (Fig. 1).

## Discussion and Conclusion

In view of the above‐mentioned results, we conclude that the mass percentages of arginine‐rich MPP in their conjugates are critically important for the life security of the mice, and the arginine‐rich MPPs maybe more suitable for using in the macro‐molecular conjugates, but not in the small‐molecular ones.

## Disclosures

None declared.
